# Increased Risk of Common Orthopedic Surgeries for Patients with Rheumatic Diseases in Taiwan

**DOI:** 10.3390/medicina58111629

**Published:** 2022-11-11

**Authors:** Min-Chih Hsieh, Malcolm Koo, Chia-Wen Hsu, Ming-Chi Lu

**Affiliations:** 1 Division of Obstetrics and Gynecology, Dalin Tzu Chi Hospital, Buddhist Tzu Chi Medical Foundation, Dalin, Chiayi 622401, Taiwan; 2 Graduate Institute of Long-term Care, Tzu Chi University of Science and Technology, Hualien City 970302, Taiwan; 3 Dalla Lana School of Public Health, University of Toronto, Toronto, ON M5T 3M7, Canada; 4 Department of Medical Research, Dalin Tzu Chi Hospital, Buddhist Tzu Chi Medical Foundation, Dalin, Chiayi 622401, Taiwan; 5 Division of Allergy, Immunology and Rheumatology, Dalin Tzu Chi Hospital, Buddhist Tzu Chi Medical Foundation, Dalin, Chiayi 622401, Taiwan; 6 School of Medicine, Tzu Chi University, Hualien City 970374, Taiwan

**Keywords:** rheumatoid arthritis, systemic lupus erythematosus, ankylosing spondylitis, psoriasis, total knee replacement, total hip replacement, spine surgery

## Abstract

*Background and Objectives:* Rheumatic diseases, including rheumatoid arthritis, ankylosing spondylitis, psoriasis, and systemic lupus erythematosus (SLE), are characterized by chronic arthritis or spondyloarthritis, which can lead to joint and spine destruction. Our previous studies showed that the risk of common orthopedic surgeries, including total knee replacement (TKR), total hip replacement (THR), or spine surgery, was increased in patients with rheumatoid arthritis, ankylosing spondylitis, psoriasis, and SLE. The aim of this review was to summarize the risk of TKR, THR, cervical spine, and lumbar spine surgery on the basis of studies conducted using data from Taiwan’s National Health Insurance Research Database (NHIRD). *Materials and Methods:* The risk of TKR, THR, cervical spine surgery, and lumbar spine surgery in patients with rheumatoid arthritis, ankylosing spondylitis, psoriasis, and SLE was summarized from the results of our previous studies and unpublished findings based on NHIRD data. *Results:* Patients with rheumatoid arthritis and psoriasis and men with ankylosing spondylitis showed an increased risk of TKR. Patients with rheumatoid arthritis, ankylosing spondylitis, and women with SLE showed an increased risk of receiving THR. Only patients with ankylosing spondylitis had an increased risk of cervical spine surgery, and patients with rheumatoid arthritis or ankylosing spondylitis showed an increased risk of lumbar spine surgery. Although the risk of THR, TKR, or spine surgery in these patients has declined in the era of biologics use, direct evidence for the effects of biologics agents is not yet available. *Conclusions:* There was an increased risk of common orthopedic surgery in patients with rheumatoid arthritis, ankylosing spondylitis, psoriasis, and SLE. Clinicians should be vigilant to reduce the increased risk of TKR and THR in young and middle-aged patients with rheumatoid arthritis, THR in young patients with ankylosing spondylitis, and young female patients with SLE, as well as cervical spine surgery in young patients with ankylosing spondylitis.

## 1. Introduction

Rheumatic diseases are a group of diseases characterized by chronic joint inflammation, leading to the destruction of the joints and spine. Rheumatic diseases are the major cause of disability worldwide, and the burden of rheumatic diseases is increasing [1]. Rheumatoid arthritis, ankylosing spondylitis, psoriasis, and systemic lupus erythematosus (SLE) are common rheumatic diseases that cause active arthritis and can lead to joint deformity. 

Rheumatoid arthritis is a common systemic autoimmune disease characterized by chronic inflammation of the peripheral joints. Chronic inflammation in the peripheral joints can lead to joint destruction that results in discomfort and disability. In addition to the peripheral joints, rheumatoid arthritis can involve large joints, including the knee, hip, and cervical [2,3] or lumbar spine [4,5]. The prevalence of rheumatoid arthritis ranges from 0.5 to 1.0% around the world, with a female-to-male ratio of 2.5:1. Rheumatoid arthritis commonly occurs in people aged 40–70 years, with the incidence increasing with age [6]. The prevalence of rheumatoid arthritis in Taiwan was found to range from 0.26% to 0.93% [7], and patients with rheumatoid arthritis were associated with a higher mortality rate compared with controls [8].

Ankylosing spondylitis belongs to the spondyloarthritis family and is characterized by a bony fusion of the vertebral joints. The prevalence of ankylosing spondylitis ranged from 0.19% to 0.54% in Taiwan (7), with a male-to-female ratio of 2:1 [9]. Because of long-standing inflammation of the spine, patients with ankylosing spondylitis often develop spinal deformities that lead to spine instability and neurological deficits. Ankylosing spondylitis can also affect peripheral large joints, including knees and hips [10].

Psoriasis is a common chronic, immune-mediated skin disease presented as erythematous, thick, and scaly areas of the skin [11]. The prevalence of psoriasis was estimated to vary from 0.16% to 0.23% in Taiwan [12]. Around 20%–30% of patients with psoriasis could develop psoriatic arthritis [13], which can cause joint damage leading to deformity and may require surgery to alleviate pain and restore function [14]. However, a study based on the Taiwan National Health Insurance Research Database (NHIRD) showed that 8.2% of patients with psoriasis had psoriatic arthritis [15], and therefore, increased effort should be made to improve the diagnosis of psoriatic arthritis. Psoriatic arthritis can affect the spine, causing inflammatory neck and back pain, eventually leading to reduced spinal mobility [16].

SLE is a prototype of the systemic autoimmune disease, and it predominately affects women during their childbearing age [17]. The prevalence of SLE was 14.3 per 10,000 people in the female population in 2011 in Taiwan [18]. SLE typically involves the joints, skin, kidneys, lungs, nerve systems, and hematological systems. Patients with SLE showed increased morbidity and mortality. In the past, joint involvement in SLE was considered mild and only caused pain in the peripheral joints. However, current evidence shows that patients with SLE can have active, erosive arthritis, which leads to the deformity of joints [19,20]. Mertelsmann-Voss et al. reported that patients with SLE had an increased risk of receiving arthroplasty on the hip and knee joints in the United States [21].

## 2. Common Orthopedic Surgeries

Both total knee replacement (TKR) and total hip replacement (THR) are common orthopedic surgery for severe joint destruction from osteoarthritis, rheumatic diseases, or osteonecrosis, and their rates are increasing around the world [22,23]. In Taiwan, the rate of primary TKR was 28.5 per 100,000 people in 1998 and has increased to 56.8 per 100,000 people in 2009. The rate of primary THR was 17.5 per 100,000 people in 1998 and increased to 19.5 per 100,000 people in 2009 [24]. In addition, there was a high prevalence of spinal surgeries in Taiwan, and the common spine surgeries were discectomy, laminectomy, spinal fusion, and spinal fracture reduction [25,26]. Since rheumatic diseases are characterized by active inflammation of the joints or the spine, it is expected that patients with these rheumatic diseases might show an increased risk of receiving a joint replacement or spinal surgery. Our research group had previously published several articles on the risk of THR and TKR in patients with ankylosing spondylitis, psoriasis, and SLE [27,28,29] and the risk of spinal surgery in patients with rheumatoid arthritis and ankylosing spondylitis [30,31]. 

The aim of this review was to summarize our previous study results on the risks of TKR, THR, and cervical spine and lumbar surgery. All our studies were based on data from the NHIRD in Taiwan. We also included the results of unpublished data exploring the risk of spinal surgery in patients with SLE and psoriasis. Because the risk of spinal surgery did not differ in patients with SLE or psoriasis compared with the controls, the results of these analyses were not previously published. Although Lee et al. reported that patients with rheumatoid arthritis were 4.82 times more likely to receive THR (95% confidence interval [CI] 3.84–6.04) and 3.85 times more likely to undergo TKR (95% CI 3.48–4.25) compared with controls, risks of TKR and THR in patients with RA stratified by age or sex were unavailable in their report [32]. Therefore, in the present study, our own unpublished data were presented instead of those from Lee et al. for these risk estimates. As patients with SLE are predominantly female, only women were included in the analysis of SLE.

## 3. Risk of Total Knee Replacement in Patients with Rheumatic Diseases

Among patients with rheumatic diseases, the risk of overall TKR was highest in patients with rheumatoid arthritis (adjusted incidence rate ratio (aIRR) = 3.77; 95% confidence interval [CI] 2.82–5.04), followed by patients with psoriasis (aIRR = 1.38; 95% CI 1.09–1.75), but the risk of TKR was not significantly elevated in patients with ankylosing spondylitis or female patients with SLE (Table 1). 

When stratified by sex, we found that both male (aIRR = 3.27; 95% CI 1.53–7.02) and female (aIRR = 3.93; 95% CI 2.87–5.39) patients with rheumatoid arthritis showed an elevated risk of receiving TKR. Only male patients showed an elevated risk of TKR (aIRR = 1.89; 95% CI 1.04–3.41), and female patients with psoriasis showed an elevated risk of TKR (aIRR = 1.44; 95% CI 1.08–1.93). 

As for the effect of age, all age groups showed an increased risk of TKR in patients with rheumatoid arthritis. It is an unexpected finding that young patients with rheumatoid arthritis (20–44 years) showed a very high risk of receiving TKR (aIRR = 74.18; 95% CI 9.80–561.38). In psoriasis, the older age group (60–80 years) showed a significantly elevated risk (aIRR = 1.31; 95% CI 1.00–1.71) of receiving TKR. 

Currently, the use of biologics along with early, aggressive treatment strategies has allowed patients with rheumatoid arthritis to better control their disease activities. The risk of receiving TKR and THR in patients with rheumatoid arthritis has decreased after the start of the era of biologics agents in Japan and Canada [33,34]. Finally, in patients with ankylosing spondylitis or SLE, the risk of TKR was not elevated when stratified by age group.

## 4. Risk of Total Hip Replacement in Patients with Rheumatic Diseases

The risk of THR was the highest in female patients with SLE (aIRR = 6.47; 95% CI 2.43–17.22), followed by patients with ankylosing spondylitis (aIRR = 5.91; 95% CI 3.39–10.30), and patients with rheumatoid arthritis (aIRR = 3.30; 95% CI 1.95–5.60) (Table 2). The risk of receiving THR did not increase in patients with psoriasis. 

When stratified by sex, both male (aIRR = 4.35; 95% CI 1.69–11.23) and female (aIRR = 2.86; 95% CI 1.50–30.18) patients with rheumatoid arthritis showed an increased risk of receiving THR. In patients with ankylosing spondylitis, only male patients showed an increased risk of receiving THR. 

As for the effect of age, both the young (aIRR = 6.96; 95% CI 1.61–30.18) and middle age (aIRR = 7.00; 95% CI 2.78–17.62) group patients with rheumatoid arthritis showed an elevated risk of receiving THR. In patients with ankylosing spondylitis, both the younger and older age groups showed an elevated risk of receiving THR, and the risk of THR in the young age group (20–39 years) was very high (aIRR = 27.66; 95% CI 6.13–124.81). In SLE, the younger age group (20–44 years) also showed an increased risk of THR (aIRR = 7.70; 95% CI 2.19–27.12), and the main cause of THR was osteonecrosis. The main reason for osteonecrosis in patients with SLE was high-dose steroid usage. Therefore, rheumatologists should be vigilant regarding the use of steroids for SLE treatment. 

In the era of biologics use, the risk of THR has begun to decrease in patients with rheumatoid arthritis [33,34]. For patients with ankylosing spondylitis, the need for THR has also changed [35] and decreased in those under 60 years of age [36]. However, Stovall et al. indicated that the risk of THR/TKR was not reduced with any combinations of NSAIDs, DMARDs, or tumor necrosis factor inhibitor (TNFi) in people with ankylosing spondylitis or psoriatic arthritis [37]. In patients with rheumatoid arthritis, the usage of TNFi was only associated with a reduction in risk for THR in those over 60 years old [38]. Therefore, there are still debates over the main cause of the decreased risk of THR/TKR in recent years.

## 5. Risk of Cervical Spine Surgery in Patients with Rheumatic Diseases

The risk of cervical spine surgery was only increased in patients with ankylosing spondylitis (aIRR = 2.36; 95% CI 1.55–3.59) (Table 3). When stratified by sex and age, only male (aIRR = 2.92; 95% CI 1.68–5.08) patients with ankylosing spondylitis showed an increased risk of receiving cervical spine surgery. Both the younger age group (aIRR = 5.75; 95% CI 2.08–15.86) and the middle age group (aIRR = 2.91; 95% CI 1.63–5.20) showed an increased risk of receiving cervical spine surgery in patients with ankylosing spondylitis. Although patients with rheumatoid arthritis are known to have cervical spine involvement, we did not find an increased risk of receiving cervical spine surgery in our cohort. A reason for this could be that the relative mean follow-up period was too short (only 6.0 years) in our patients with rheumatoid arthritis [39,40].

## 6. Risk of Lumbar Spine Surgery in Patients with Rheumatic Diseases

Both the patients with rheumatoid arthritis (aIRR = 2.14; 95% CI 1.46–3.15) and ankylosing spondylitis (aIRR = 2.33; 95% CI 1.85–2.93) showed an increased risk of lumbar spine surgery (Table 4). When stratified by sex, only female (aIRR = 2.44; 95% CI 1.61–3.69) patients with rheumatoid arthritis showed an increased risk of receiving lumbar spine surgery. Both male (aIRR = 2.13; 95% CI 1.53–2.96) and female (aIRR = 2.53; 95% CI 1.84–3.49) patients with ankylosing spondylitis showed an increased risk of receiving lumbar spine surgery. 

When stratified by age, patients with rheumatoid arthritis in the middle (45–59 years) (aIRR = 2.32 95% CI 1.30–4.13) and old age group (59–80) (aIRR = 1.90; 95% CI 1.10–3.29) showed a higher risk of receiving lumbar spine surgery. On the other hand, in patients with ankylosing spondylitis, all three age groups showed an increased risk of receiving lumbar spine surgery (20–39 years: aIRR = 3.14; 95% CI 1.91–5.18; 40–59 years: aIRR = 2.43; 95% CI 1.72–3.43); 60–80 years: aIRR = 1.75; 95% CI 1.18–2.59). Generally, male patients with ankylosing spondylitis have more severe radiographic changes in the spine [41]. However, our study also showed an increased risk of lumbar spine surgery in female patients with ankylosing spondylitis. Therefore, clinicians should also be vigilant for the possibility of lumbar spine disorder in female patients with ankylosing spondylitis. The cause of increased risk for spinal surgery in patients with ankylosing spondylitis might be related to the disease manifestation itself. 

## 7. Summary

The risk of receiving TKR was increased in patients with rheumatoid arthritis, psoriasis, and male patients with ankylosing spondylitis. On the other hand, the risk of receiving THR was increased in patients with rheumatoid arthritis, ankylosing spondylitis, and women with SLE. Patients with ankylosing spondylitis also showed a higher risk of cervical and lumbar spine surgery because of the nature of the disease itself. Moreover, patients with rheumatoid arthritis showed an increased risk of receiving lumbar spine surgery. Recent studies suggested that the trend for orthopedic surgery has declined in TKR and THR in rheumatoid arthritis, as well as in THR among patients with ankylosing spondylitis. The use of biologics for treating rheumatic diseases has been considered a key factor in reducing the risk of orthopedic surgery. However, direct evidence is still lacking. Physicians should be aware of the possibility of the knee, hip, and spinal destruction in patients with rheumatic diseases. Action should be taken to reduce the increased risk of receiving TKR in young patients with rheumatoid arthritis, receiving THR in patients with ankylosing spondylitis and female patients with SLE, and receiving cervical spine surgery in young patients with ankylosing spondylitis.

## Figures and Tables

**Table 1:** A summary of the incidence rate ratio, 95% confidence interval, and *p* value of total knee replacement surgery in patients with rheumatoid arthritis, ankylosing spondylitis, psoriasis, and systemic lupus erythematosus.

	Rheumatoid Arthritis (*n* = 1557)	Ankylosing Spondylitis [28] (*n* = 3462)	Psoriasis [27] (*n* = 10,819)	Systemic Lupus Erythematosus [29] * (*n* = 557)
Overall	3.77 (2.82–5.04) <0.001	1.10 (0.78–1.54) 0.591	1.38 (1.09–1.75) 0.007	NA
Male	3.27 (1.53–7.02) 0.002	1.89 (1.04–3.41) 0.036	1.29 (0.87–1.92) 0.209	NA
Female	3.93 (2.87–5.39) <0.001	0.88 (0.59–1.34) 0.554	1.44 (1.08–1.93) 0.014	1.81 (0.69–4.75) 0.227
Age effect (only the significant age interval was shown)	20–44 years 74.18 (9.80–561.38) <0.00145–59 years 6.86 (4.20–11.20) <0.001 60–80 years 1.68 (1.08–2.62) 0.02	NS	60–80 years 1.31 (1.00–1.71) 0.047	NS

Data were presented as an incidence rate ratio (IRR) and 95% confidence interval (CI), and *p* value; NA—not available; NS—no statistically significant association; * For systemic lupus erythematosus, only female patients were analyzed.

**Table 2:** A summary of the incidence rate ratio, 95% confidence interval, and *p* value of total hip replacement surgery in patients with rheumatoid arthritis, ankylosing spondylitis, psoriasis, and systemic lupus erythematosus.

	Rheumatoid Arthritis (*n* = 1287)	Ankylosing Spondylitis [28] (*n* = 3462)	Psoriasis [27] (*n* = 10,819)	Systemic Lupus Erythematosus [29] * (*n* = 557)
Overall	3.30 (1.95–5.60) <0.001	5.91 (3.39–10.30) <0.001	1.27 (0.88–1.84) 0.204	NA
Male	4.35 (1.69–11.23) 0.002	12.59 (5.54–28.58) <0.001	1.40 (0.90–2.19) 0.137	NA
Female	2.86 (1.50–30.18) 0.001	2.34 (0.95–5.73) 0.064	1.09 (0.55–2.19) 0.803	6.47 (2.43–17.22) <0.001
Age effect (only showed the significant age interval)	20–44 years 6.96 (1.61–30.18) 0.010 45–59 years 7.00 (2.78–17.62) <0.001	20–39 years 27.66 (6.13–124.81) <0.00140–80 years 3.84 (2.00–7.36) <0.001	NS	20–44 years 7.70 (2.19–27.12) 0.001

Data were presented as an incidence rate ratio (IRR) and 95% confidence interval (CI), and *p* value; NA—not available; NS—no statistically significant association; * For systemic lupus erythematosus, only female patients were analyzed.

**Table 3:** A summary of the incidence rate ratio, 95% confidence interval, and *p* value of cervical spine surgery in patients with rheumatoid arthritis, ankylosing spondylitis, psoriasis, and systemic lupus erythematosus.

	Rheumatoid Arthritis [31] (*n* = 1287)	Ankylosing Spondylitis [30] (*n* = 3462)	Psoriasis (*n* = 10,677)	Systemic Lupus Erythematosus * (*n* = 471)
Overall	1.79 (0.68–4.71) 0.238	2.36 (1.55–3.59) <0.001	1.10 (0.74–1.65) 0.638	NA
Male	0.89 (0.11–7.44) 0.915	2.92 (1.68–5.08) <0.001	1.14 (0.71–1.84) 0.590	NA
Female	2.27 (0.74–6.98) 0.153	1.78 (0.92–3.44) 0.087	1.01 (0.47–2.16) 0.991	1.55 (0.31–7.78) 0.596
Age effect (only showed the significant age interval)	NS	20–39 years 5.75 (2.08–15.86) 0.001 40-59 years 2.91 (1.63–5.20) <0.001	NS	NS

Data were presented as an incidence rate ratio (IRR) and 95% confidence interval (CI), and *p* value; NA—not available; NS—no statistically significant association; * For systemic lupus erythematosus, only female patients were analyzed.

**Table 4:** A summary of the incidence rate ratio, 95% confidence interval, and *p* value of lumbar spine surgery in patients with rheumatoid arthritis, ankylosing spondylitis, psoriasis, and systemic lupus erythematosus.

	Rheumatoid Rthritis [31] (*n* = 1287)	Ankylosing Spondylitis [30] (*n* = 3462)	Psoriasis (*n* = 10,677)	Systemic Lupus Erythematosus * (*n* = 471)
Overall	2.14 (1.46–3.15) <0.001	2.33 (1.85–2.93) <0.001	1.09 (0.89–1.34) 0.393	NA
Male	0.99 (0.32–3.05) 0.989	2.13 (1.53–2.96) <0.001	1.05 (0.80–1.38) 0.710	NA
Female	2.44 (1.61–3.69) <0.001	2.53 (1.84–3.49) <0.001	1.16 (0.85–1.58) 0.351	0.27 (0.04–1.99) 0.197
Age effect (only showed the significant age interval)	45–59 years 2.32 (1.30–4.13) 0.004 59–80 years 1.90 (1.10–3.29) 0.022	20–39 years 3.14 (1.91–5.18) <0.001 40–59 years 2.43 (1.72–3.43) <0.001 60–80 years 1.75 (1.18–2.59) 0.005	NS	NS

Data were presented as IRR (95% CI) and *p* value; NA—not available; NS—no statistically significant association; * In patients with SLE, we only included female patients.

## Data Availability

Not applicable.
